# Nanoscale compositional mapping of cells, tissues, and polymers with ringing mode of atomic force microscopy

**DOI:** 10.1038/s41598-017-12032-z

**Published:** 2017-09-19

**Authors:** M. E. Dokukin, I. Sokolov

**Affiliations:** 1 Department of Mechanical Engineering, Tufts University, Medford, MA USA; 2 Department of Biomedical Engineering, Tufts University, Medford, MA USA; 3 Department of Physics, Tufts University, Medford, MA USA

## Abstract

Recently developed sub-resonance tapping modes (such as Digital Pulse, Peak Force Tapping, HybriD, etc.) of atomic force microscopy (AFM) allow imaging of compositional contrast of (bio)materials and biological cells down to the nanoscale. Here we report on a powerful extension of those modes, “ringing” mode, which more than doubles the number of non-trivial physical channels that can be collected with a regular sub-resonance tapping. It can simultaneously record five new additional compositional parameters related to adhesive and viscoelastic properties of the sample surface: the restored (averaged) adhesion, adhesion height, pull-off neck height, detachment distance, and detachment energy losses. Ringing mode can be up to 20 times faster and showing fewer artifacts compared to the existing sub-resonance tapping modes. Ringing mode is based on an analysis of ringing signal of the AFM cantilever after detaching the AFM probe from the sample surface (this signal is currently treated as noise, and typically filtered out in the existing modes). We demonstrate that this new mode allows recording robust and unique information on fixed human epithelial cells, corneocyte skin flakes, and polymers used for bioimplants.

## Introduction

Traditional atomic force microscopy (AFM) is used to visualize sample surfaces, including biomaterials, living and fixed cells down to the nanoscale^1,2^. It is important because most of biomaterials and cells are heterogeneous at that scale. It is informative to obtain a compositional contrast of such samples^3^. There have been a number of modes developed, from various resonance methods (such as tapping^4^, multifrequency^5,6^, contact resonance^7,8^, etc.) to non-resonance ramping modes (such as Force-Volume-, Pulse-Force-, Jump- modes, and recently, Digital Pulse-Force, HarmoniX, PeakForce Tapping™, HybriD, AM-FM Viscoelastic Mapping, QI™ modes). While the comparison of these modes is beyond the scope of this work, resonance and non-resonance modes typically provide complementary information about the sample. All these modes are based on the analysis of viscous, elastic, and adhesive properties of the sample surface. Although it is possible to analyze some long-range forces, such as electrostatic and steric, it has been done only with slow force-volume^9,10^ and lift^11,12^ modes so far.

Being a relatively slow scanning technique, AFM is rather fast in collecting a large amount of information per second^13^. It allows for simultaneous recording of multiple channels of information. For example, the sub-resonance ramping modes allow recording of the sample height, adhesion, sample surface stiffness, viscoelastic dissipation energy. Such information is important for biomedical applications. It has been found that cell mechanics is a new biophysical parameter correlated with many human diseases^14–16^ and even aging^17,18^. Mechanical cues of the cell environment define the cell fate and phenotype^19,20^. It was demonstrated that the adhesion AFM imaging channel allows virtually ambiguous segregation of malignant and normal human cervical epithelial cells^21,22^.

In all sub-resonance modes, the AFM probe ramps vertically (while the sample moves in lateral direction) oscillating at the frequencies much smaller than the resonant frequency of the AFM cantilever. These modes have been introduced to combine quantitative advantages of the Force-Volume mode while retaining the fast speed of typical imaging scanning. Although quantitative potential of these modes was demonstrated^23^, a slow scanning speed still limits their use in many practical applications. As was mentioned^21^, imaging of human cervical epithelial cells with HarmoniX and PeakForce Tapping allowed to segregate cancerous and normal cells with unprecedented accuracy. However, it took about 1.5 hours to obtain a map of one cell, which makes it impractical for a potential clinical use. In addition, some particular orientations of the semi-transparent samples can create interference artifacts which are a serious issue for quantitative interpretation of results.

**Figure 1:**
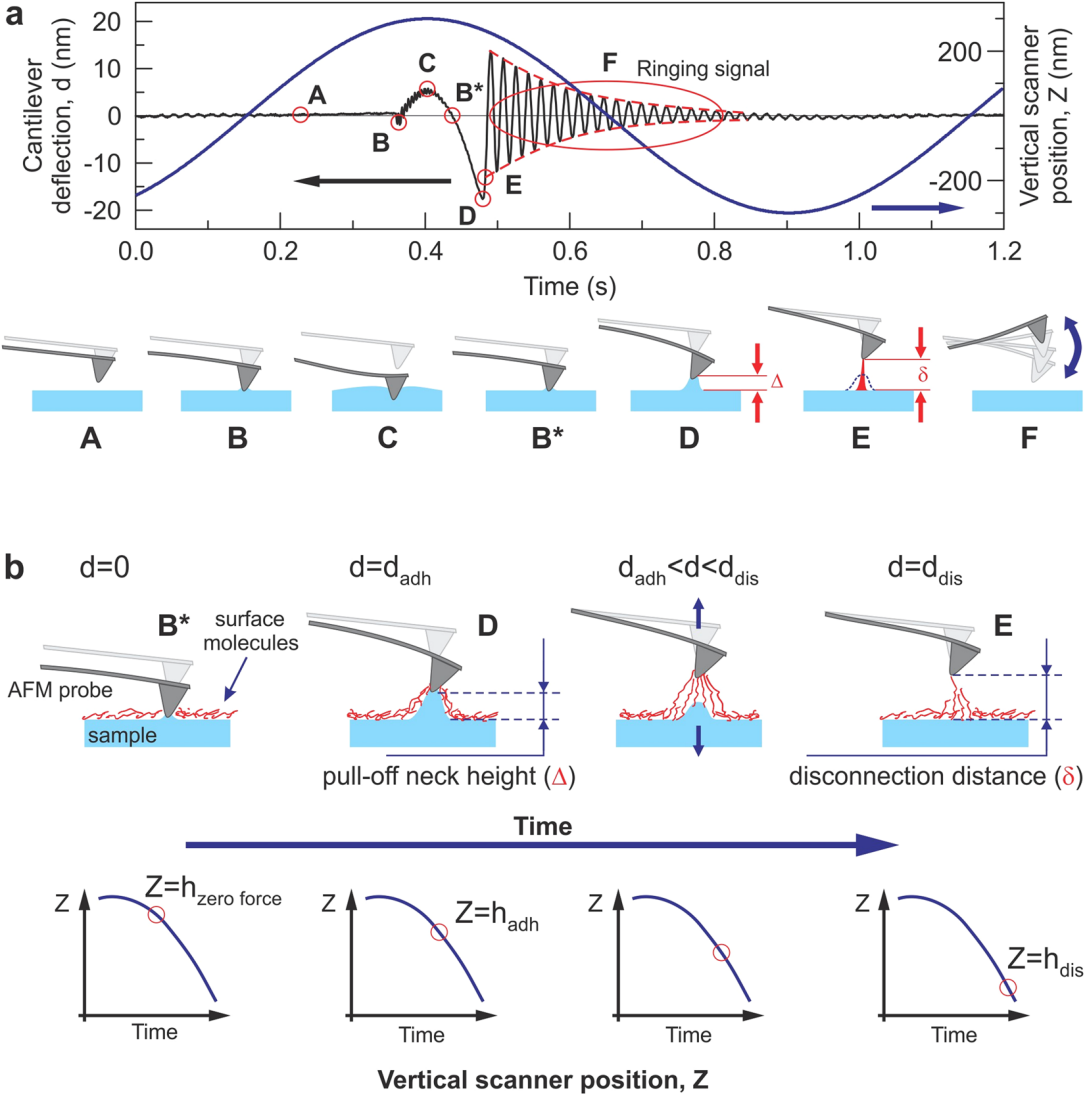
A schematic of the signal exploited in ringing mode. The cantilever deflection *d* and vertical position of the AFM scanner *Z* versus time are shown. (**a**) One full cycle of 1 kHz vertical oscillation of the Z scanner is presented. A typical unfiltered signal of the cantilever deflection *d* (or force = *kd*, where *k* is the spring constant of the cantilever) as a function of time is shown. The dash-envelope lines show the decrease of the oscillating amplitude of the AFM cantilever due to dissipation. Positive values of the cantilever deflection *d* are due to forces of repulsion, and vice versa, and negative values stand for the attraction between the AFM probe and sample surface. Specific points during the cantilever motion indicate the following probe positions: (A) far from the sample surface, (B) touches the sample, (C) deforms the sample surface, (B*) touches the surface with zero force when retracting, (D) starts a fast detachment (pull-off) from the sample (the point defining adhesion force), (E) completely detaches from the sample (the point defining the restored adhesion force), (F) starts free oscillations above the sample surface. (**b**) Definition of the neck height Δ, the size of the neck of the pull-off deformation caused by the AFM probe right before disconnection from the surface; definition of disconnection distance δ, the size of the molecular tails pulled from the surface by the AFM probe during the disconnection from the sample surface.

Here we introduce a new “ringing” mode, which is based on the extraction of useful information from ringing signal of the AFM probe after it disconnects from the sample surface, Fig. 1. This ringing part of the deflection signal is currently treated as noise and filtered out (or ignored) in the existing sub-resonance tapping modes. To get ringing signal, either filtering of the signal should be switched off within the AFM control software or the unfiltered signal can be recorded directly. The unfiltered signal can then be processed either in real time or later, offline. Our technique adds five new channels of information to the existing multiple channels of non-resonance ramping modes, allows for up to 20 times faster imaging, and shows less artifacts.

## Results

### Basics of ringing mode

Here we describe a new method to process raw signals recorded by AFM, which operates in one of the sub-resonance tapping modes. We demonstrate realization of this method using an example of PeakForce Tapping mode (sub-resonance mode Bruker-Nano; details of the implementation are described in Supplementary Note 1). In sub-resonance tapping modes, the AFM cantilever oscillates vertically above a sample surface so that the AFM probe touches the sample, deforms it, and disconnects within each oscillation, Fig. 1a. A lateral (X,Y) slow scanning of the sample surface continues during such oscillations. The maximum deflection of the cantilever at each contact is kept constant by the AFM feedback system during the scanning. The AFM deflection signal is recorded and processed in real time. It brings the maps of sample height, values of the pull-off (adhesion) force, mechanical stiffness, and viscoelastic losses during the sample deformation by the AFM probe at each cycle.

The basic feature of ringing mode is the use information stored in ringing signal, resonance oscillations of the AFM cantilever after the probe detached from the sample surface. An example of typical unfiltered deflection of the AFM cantilever is shown in Fig. 1a. One can see ringing motion of the cantilever after detachment from the sample surface (regio﻿n F﻿ in Fig. 1a), which happens at the resonance frequency of the cantilever (~75 kHz in this example). This signal is typically treated either as parasitic or noise in the regular sub-resonance modes, and consequently, filtered out or just ignored. However, this signal contains useful information, analysis of which allows one to introduce several new physical properties that can be measured, and consequently, imaged. It is described in the next sections.

### Imaging of the (averaged) restored adhesion force

Here we introduce a new surface property parameter, which can be visualized in ringing mode, the averaged restored adhesion. Figure 1 graphically defines this property. After the pull-off (point D in Fig. 1), the AFM cantilever jumps away from the surface, and the ringing starts. Each oscillation has smaller and smaller amplitude due to dissipation. Technically, this can be used to calculate the quality factor. However, this value, as well as the spring constant can be measured separately before the scanning during the calibration process. (see, the Methods section for more detail). When knowing the quality factor, one can extrapolate the value of the adhesion force to the moment of pull-off or more precisely, to the time when the extrapolation curve crosses the deflection curve. This is shown in Fig. 1a as point E. This is what we call the restored adhesion (note, not yet averaged).

It should be understood that the restored and regular adhesions are different physical characteristics. The difference between the restored and regular adhesion comes from the molecular tails detaching from the AFM probe after it lost the adhesive contact with the sample surface. It results in an additional loss of energy dissipated during the disconnection. Mathematically, the value of the restored adhesion can be found, in principle, from any point of the ringing signal. Because the measured values of the cantilever deflection have an intrinsic noise, it is advantageous to calculate the restored adhesion using multiple points of ringing signal, and subsequently, to average the obtained values to decrease the noise in the measured value of the restored adhesion. Equation (1) gives the mathematical definition of the *averaged* restored adhesion force ${\bar{F}}_{Ra}$ (the bar stands for the averaged value):

1 $${\bar{{F}}}_{{Ra}}=k{d}_{Ra}=k\frac{1}{N}\sum _{k=1}^{N}\frac{d({t}_{k})}{Exp(-\pi {f}_{0}{t}_{k}/Q)|Sin(2\pi {f}_{0}{t}_{k}+\phi )|},$$

where *k* in the spring constant of the AFM cantilever, *d*_*Ra*_ is the cantilever deflection at the time of detachment, *d(t*_*k*_) is the cantilever deflection recorded at time *t*_*k*_, *f*_0_ is the resonance frequency of the AFM cantilever, φ is the phase (zero at a maximum of the ringing signal), *Q* is the quality factor of the cantilever.

**Figure 2:**
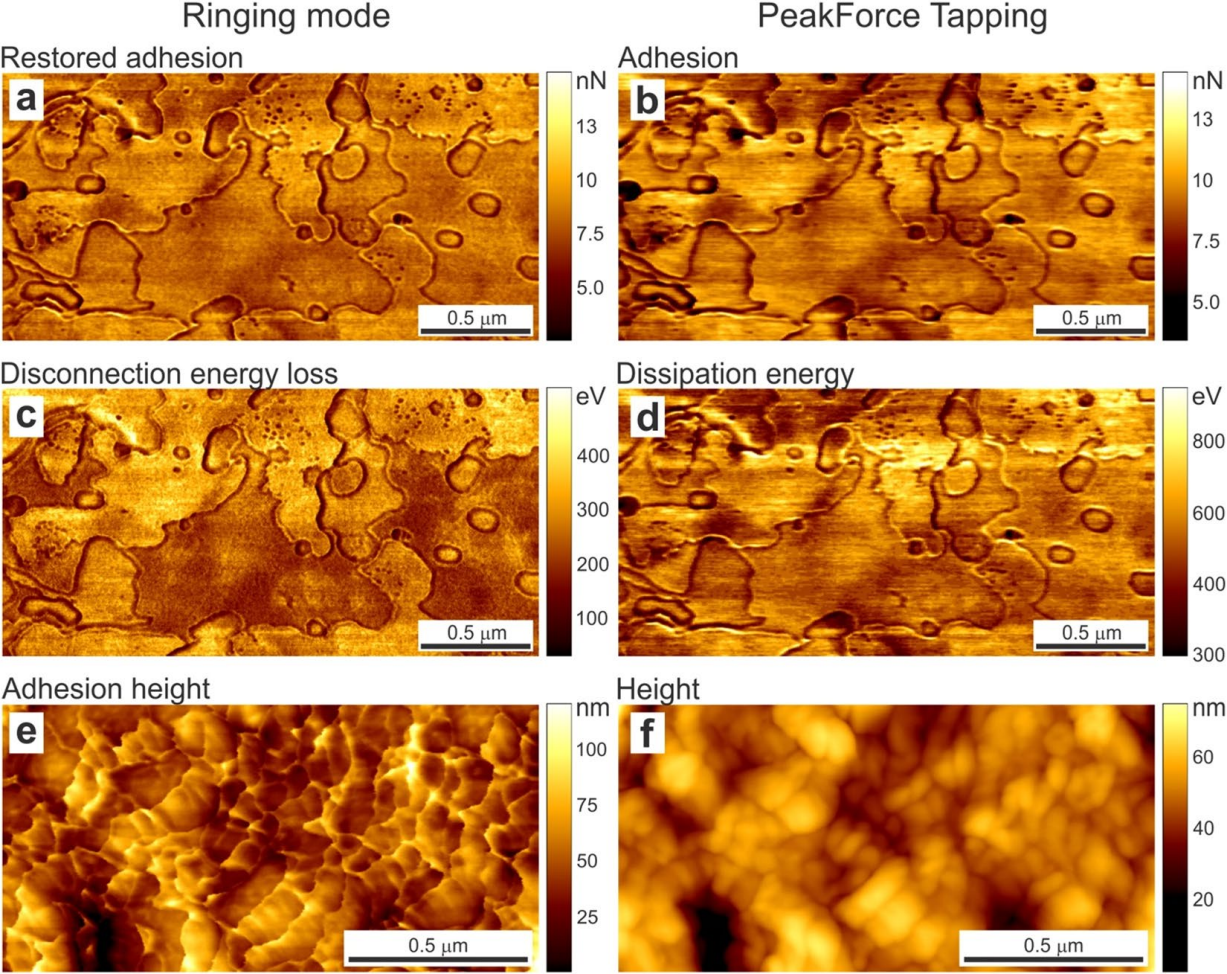
Examples of new channels of ringing mode compared to the simultaneously imaged channels of PeakForce tapping that present quantities of the same dimension (though having different physical meaning). AFM maps of human skin flake surfaces (**a**–**d**) and A375 human melanoma cells (**e**,**f**) simultaneously imaged in the new ringing (left column) and existing PeakForce Tapping (right column) modes. The scan rate is 0.5 Hz (2 sec per line). Newly introduced surface properties are shown in the left column, (**a**,**c**,**e**). The maps simultaneously recorded with the regular PeakForce Tapping mode (**b**,**d**,**f**), which are remote relatives of corresponding channel of ringing mode, are presented for comparison. (**a**) The averaged restored adhesion, (**b**) the regular adhesion maps, (**c**) disconnection energy loss during separation of the AFM probe from the sample surface, (**d**) dissipation energy, (**e**) the adhesion height and (**f**) regular height recorded in the PeakForce tapping mode.

Images of the restored adhesion measured on human corneocytes are shown in Fig. 2a. The standard adhesion image recorded in PeakForce Tapping mode is shown for comparison (Fig. 2b). One can see a noticeable difference in the image resolution. It is discussed in the Advantages of ringing mode section.

### Imaging of energy losses due to the probe-sample disconnection

Here we introduce another new surface property parameter, which can be extracted from ringing signal. It is the energy losses due to dissipative disconnection of the AFM probe from the sample surface, or “*disconnection energy loss*”, *E*_*d loss*_. This energy loss comes from the energy of viscoelastic pull of the sample material under the probe and from rapturing the molecular contacts between the probe and sample. The latter can include the breakage of water meniscus if the scanning is done in air. As was shown^21,24^, the contribution from the adsorbed water layer can be ignored when measuring adhesive properties of surfaces for samples like fixed cells up to the relative humidity of 60–70%. Our analysis shows that the contribution of the capillary forces to *E*_*d loss*_ can typically be ignored for almost any reasonable set of parameters (see, Supplementary note 2 for detail; Supplementary Figure S5 can be used to estimate the energy losses due to the breakage of the capillary bridge between the AFM probe and sample surface).

*E*_*d loss*_ is defined as follows. One can see that the restored adhesion ${\bar{F}}_{Ra}=k{d}_{Ra}$ is not equal to the regular adhesion force ${F}_{a}=k{d}_{a}$ (Fig. 1, points E and D, respectively). The energy stored in the cantilever at the moment of its jump to the ringing behavior (point E) is less than the energy at the pull-off deflection (point D). This difference is *E*_*d loss*_. It equals to differences between energies stored in the cantilever when its amplitude is equal to *d*_*a*_ and *d*_*Ra*_:

2 $${E}_{dloss}=\frac{k}{2}({{d}_{a}}^{2}-{{d}_{Ra}}^{2}).$$

Images of the disconnection energy loss measured on corneocytes are shown in Fig. 2c. The regular energy dissipation channel, which is the closest parameter in the standard PeakForce Tapping mode, is shown for comparison in Fig. 2d. One can see a substantial difference between these two images. This is because the dissipation energy is calculated in the PeakForce Tapping mode as the difference between approaching and retracting trajectory of the AFM probe, which consists of a number of different contributions, the adhesion energy, the energy due to the viscoelastic response of the sample material during the contact deformation, and finally, the energy defined in our disconnection energy loss. The contribution of our new parameter is just a portion of the total energy losses (see, Supplementary note 3 for detail). As a result, one can see that the values of the disconnection energy loss are 2-3x smaller than the dissipation energy ones. Further comparison between these two energy losses is described in the Advantages of ringing mode section.

### Visualization of the sample height at the moment of pull-off (adhesion height)

The sample height at different moments of interaction between the AFM probe and sample is of interest when the sample deformation can be noticeable. For example, the sample height at the moment of maximum negative cantilever deflection (adhesion), or point D in Fig. 1, gives the image of the sample surface with no compression, but the opposite, a pull-off stretching. We suggest calling it the *adhesion height*. An example of the adhesion height map is shown in Fig. 2e. The regular height image of the same surface (recorded in PeakForce Tapping mode) is shown in Fig. 2f. One can see an obvious advantage of the adhesion height when comparing with the regular one; the latter is the position of the sample at the moment of maximum deformation (maximum positive cantilever deflection) (point C in Fig. 1). Inevitable compression of the sample surface seen in the height image does not allow to visualize fine molecular layer on the cell surface seen in Fig. 2e. At the same time, such corrugations are clearly seen in the adhesion height because the corrugations are stretched by the AFM probe at the moment of pull-off. Further comparison will be described in the Advantages of ringing mode section.

### Imaging of pull-off neck height and disconnection distance

We can now introduce two principally new channels, which have neither direct nor indirect analogy in the existing sub-resonance modes. These are the pull-off neck height and disconnection distance. The pull-off neck height is the maximum height of the neck created by the AFM probe during the probe retraction, point D in Fig. 1 (defined as Δ). The disconnection distance is defined as the size of asperities/molecules stretched by the AFM probe during the final disconnection of the probe and sample surface (between points D and E in Fig. 1, defined as δ).

From geometrical considerations shown in Fig. 1b, one can find that the pull-off neck height ${\rm{\Delta }}={h}_{zeroforce}-{h}_{adh}\,+\,$ ${d}_{adh}={h}_{zeroforce}+|{h}_{adh}-{d}_{adh}|$, where *h*_*zero force*_ is the sample height at zero force (zero deflection of the AFM cantilever, point B* in Fig. 1), *h*_*adh*_ is the adhesion height, and *d*_*adh*_ is the deflection of the cantilever at the adhesion point (D in Fig. 1). It should be noted that the sample heights and cantilever deflections are defined as positive for positive load force and negative for the negative (adhesion) ones.

**Figure 3:**
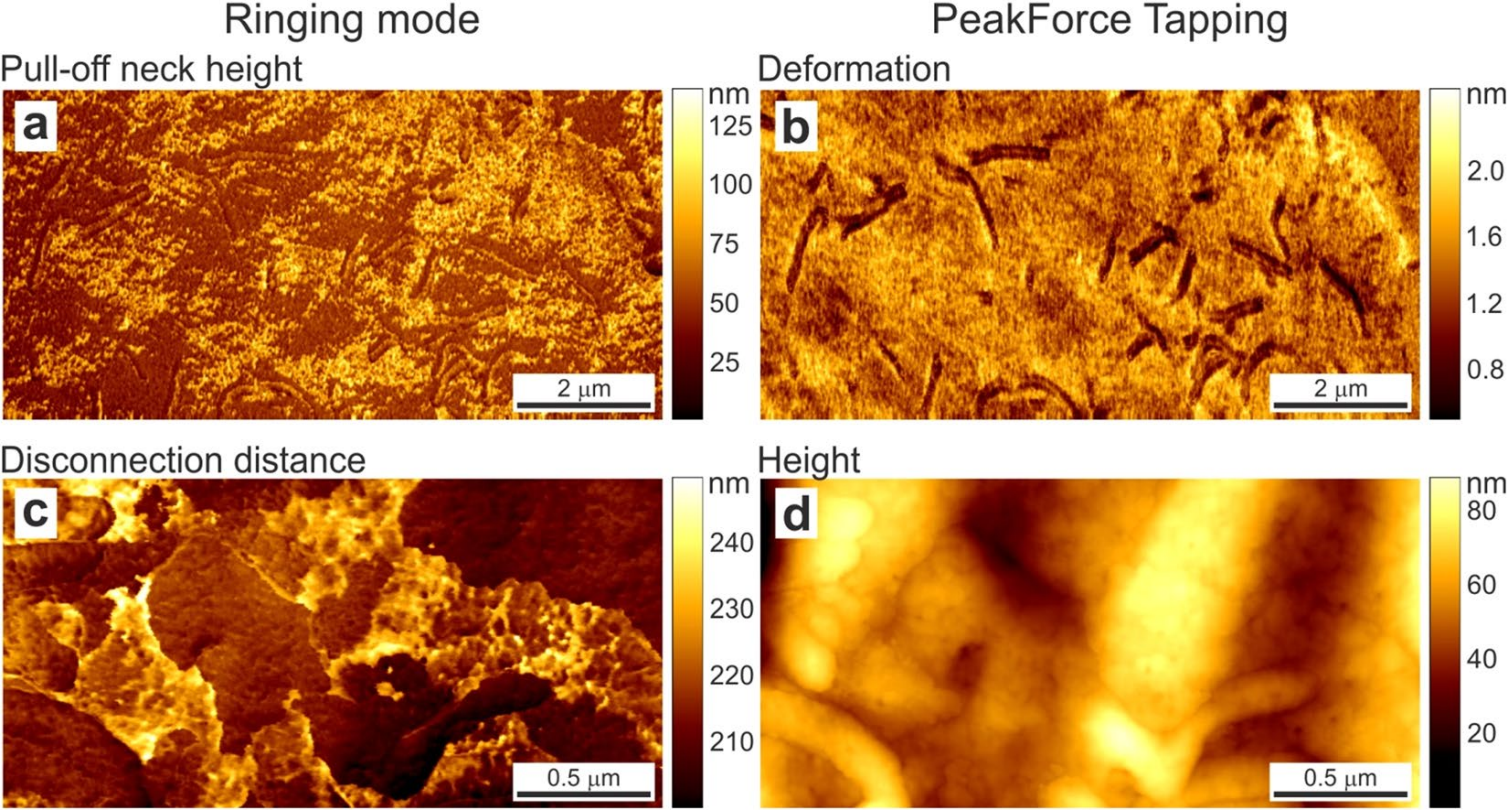
New imaging channels of ringing mode, which do not have any relatives in the regular sub- resonance tapping modes: (**a**) pull-off neck height and (**c**) disconnection distance. An example of human melanoma cell is shown. Images of surface deformation (**b**) and regular cell height (**d**) are shown as reference (simultaneously recorded in PeakForce Tapping mode). The scan rate is 0.5 Hz.

An example of pull-off neck height of a human melanoma cell is shown in Fig. 3a. It is interesting to see new complementary information, patches on the cell surface seen in Fig. 3a, whereas these patches not seen in the regular deformation map (Fig. 3b) shown for comparison. Based the observed size (40–80 nm), we can speculate that we deal with deformation of the membrane with different adhesive properties to the AFM probe (silica material).

The disconnection distance is introduced as the height of the molecular asperities/molecules stretched by the AFM probe. The final disconnection of the last stretched molecules is rather faint point in the force curve (point E in Fig. 1). With rare exceptions, it is almost impossible to see that point on the force curve. However, this point can easily be found by calculating the restored adhesion (eq. 1). From geometrical considerations, one finds that the disconnection distance can be calculated as

3 $$\begin{array}{c}\delta ={\rm{\Delta }}+({h}_{adh}-{h}_{dis})+({d}_{Ra}-{d}_{adh})=([{h}_{\mathrm{zero}\mathrm{force}}-{h}_{adh}]+{d}_{adh})+({h}_{adh}-{h}_{dis})+({d}_{Ra}-{d}_{adh})\\ \,\,\,=\,{h}_{\mathrm{zero}\mathrm{force}}-{h}_{dis}+{d}_{Ra},\end{array}$$

where *h*_*dis*_ is the sample height (vertical position of the AFM scanner) at the moment of disconnection (point E in Fig. 1).

Figure 3c shows an example of disconnection distance on a human melanoma cell. Figure 3d presents height image as a reference. One clearly sees molecular patches, which does not seem to have any correlation with the cell height. Based on the size (220–240 nm), we can speculate that these patches are presumably stretched glycocalyx. Bulky glycoproteins and glycosaccharides of this size are expected to be present on the cell surface^25^, though their patched structure was only confirmed by optical topographical SAIM images^26^. One can see that topographical information can be rather misleading for locating the glycocalyx (compare Fig. 3c and d).

### Advantages of ringing mode: Comparing the parameters visualized with ringing versus PeakForce Tapping mode

Here we compare the sample surfaces imaged simultaneously in ringing mode and in its closest relative, a standard sub-resonant tapping, PeakForce Tapping mode.

#### Averaged restored versus regular adhesion

Figure 2a and b shows a noticeably higher resolution of the averaged restored adhesion. This can be explained not only by averaging (eq. (1)) but also by the nature of definition of the regular adhesion, which requires identification of the pull-off deflection of the cantilever. Because this event is a relatively quick, the exact position of the AFM cantilever at that moment will always carry some error of digitalization.

**Figure 4:**
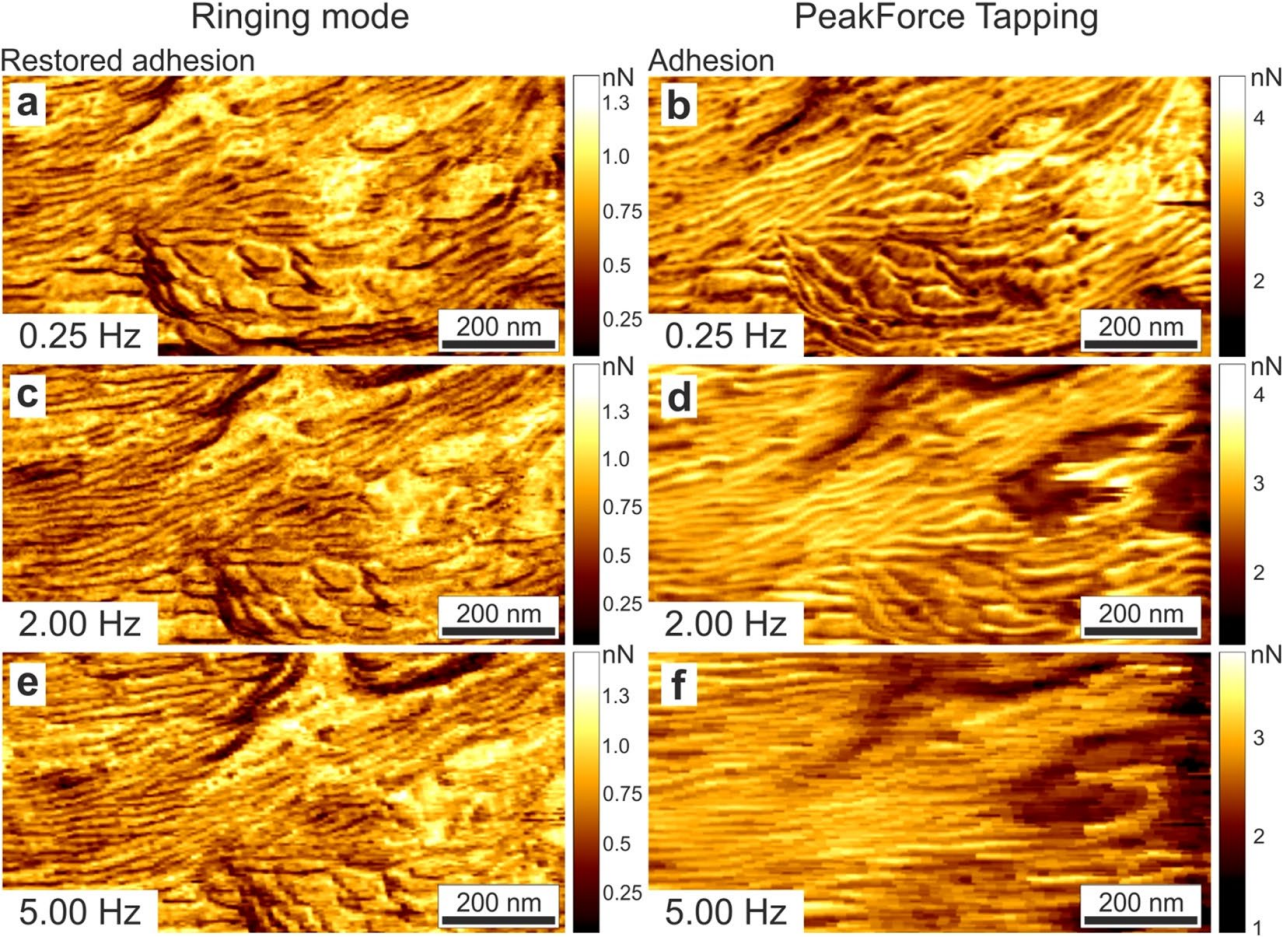
Comparison of the restored adhesion (**a**,**c**,**e**; left column) and adhesion images (**b**,**d**,**f**; right column) obtained with ringing and PeakForce Tapping modes, respectively, recorded at different scanning speeds. Maps of polystyrene-polycaprolactone composite polymeric material are shown. Lamellar structure of polycaprolactone is clearly seen in ringing mode even at 5 Hz scan speed, which is 20 times faster than a comparable quality image recorded in PeakForce Tapping. Note that a clear pixelization seen in panels (**d**) and (**f**) comes not from insufficient digital resolution (which is the same for all images) but from insufficiently fast data processing in PeakForce tapping; the same value is assigned to several pixels.

To decrease this digitalization error, multiple touch events are averaged in the existing sub-resonant modes. This results in slower imaging when using those modes. Ringing mode allows averaging within one single touch. It brings another advantage of imaging the restored adhesion, the ability to scan with a much higher speed. While ringing mode assigns values to each pixel down to a single touch, PeakForce tapping mode is not be capable of recalculating new values for each touch. It should be noted that the existing sub- resonance tapping are proprietary modes, and therefore, our explanation is based on our observations of the behavior of those modes. It is definitely possible that we missed some reasons for the faster imaging of ringing mode. Figure 4 shows the comparison of the restored adhesion and regular adhesion images recorded simultaneously at different scanning speeds. One can see that it is possible to record good images of restored adhesion without artifacts about 20x faster compared to the adhesion recorded in PeakForce Tapping mode (see, also Figure S1 for a larger number of scan speeds).

In addition to a higher resolution and speed, the images of the restored adhesion demonstrate almost no interference artifacts, which occur due to the interference of laser used to detect position of the AFM cantilever. Reflection of the laser light from the cantilever and a relatively reflective sample or substrate surface interfere with each other, thereby creating an artificial decrease or increase of the signal coming from the cantilever. This artifact is easy to recognize by a specific wavy pattering. It is strongly dependent on the sample tilt and position of the laser on the AFM cantilever. This artifact typically disappears on such samples as cells if one uses a NIR laser in the AFM detection system. Figure S2 shows simultaneously taken images of the restored and regular adhesion of a cell surface. One can clearly see the absence of interference artifacts in the restored adhesion image, Fig. S2a. The reason for the absence of the artifact is that the ringing physically happens around zero deflection of the cantilever. Therefore, the shift of the background (the reason for the artefact due to interference) can easily be identified, and subsequently, corrected.

#### Disconnection energy loss versus dissipation energy parameter

**Figure 5:**
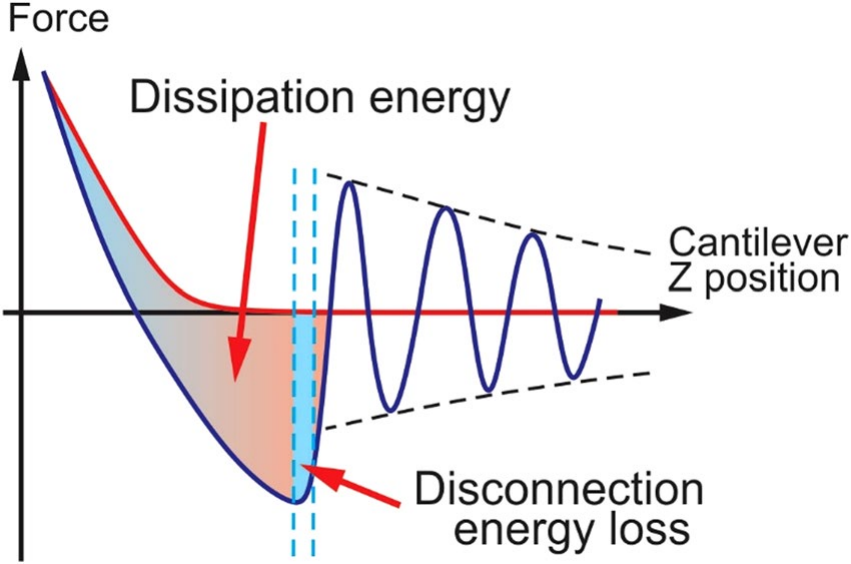
Graphical representation of dissipation energy and disconnection energy losses. Both parameters are equal to the areas between curves shown in the figure.

The dissipation energy parameter measured in standard sub-resonance tapping includes all energies lost during a vertical probe cycle, such as sample viscoelastic deformation, overcoming the force of adhesion, and the probe-surface disconnection (see, Supplementary note 3 for details). Figure 5 shows the definition of the newly introduced disconnection energy loss versus the dissipation energy. The latter is defined in PeakForce tapping mode as the area between the approach and retraction force curves. The disconnection energy losses is the area between the approaching and retracting force curves, and which is restricted by the pull off (adhesion) point and the point of disconnection between the AFM probe and sample.

Because the disconnection energy losses are accumulated only during the probe-sample detachment, this surface characteristic is much more sensitive to the dissipative long-range attraction between the AFM probe and surface, which is acting for some time after pull-off. Such an attraction can occur due to large molecules grafted to the surface (e.g., glycoproteins or polysaccharides on the cell surface) and/or pulling an easily deformable viscoelastic surface (e.g., a thin layer). An example of strong layer contrast in the disconnection energy channel is presented in Fig. 2c, in which thin layers of the surface of corneocytes are clearly seen.

It is interesting to comment on the known-artifact of sudden change of the adhesion near steps, which occurs due to the increase of contact area between the AFM probe and sample when the AFM probe touches both the step wall and the bottom part of the sample. This artifact is clearly seen in the adhesion channels, Fig. 2a,b as a line outlining thin layers of corneocytes. One can see that this artifact is much less pronounced in the disconnection energy loss channel. This is presumably due to a large difference in the disconnection energy of different layers.

Another advantage of imaging the disconnection energy is higher resolution of this channel compared to the energy dissipation, Fig. 2d. This is because the area of contact, a definitive property of resolution, is always higher in the case of regular dissipation energy.

#### Adhesion versus regular heights

**Figure 6:**
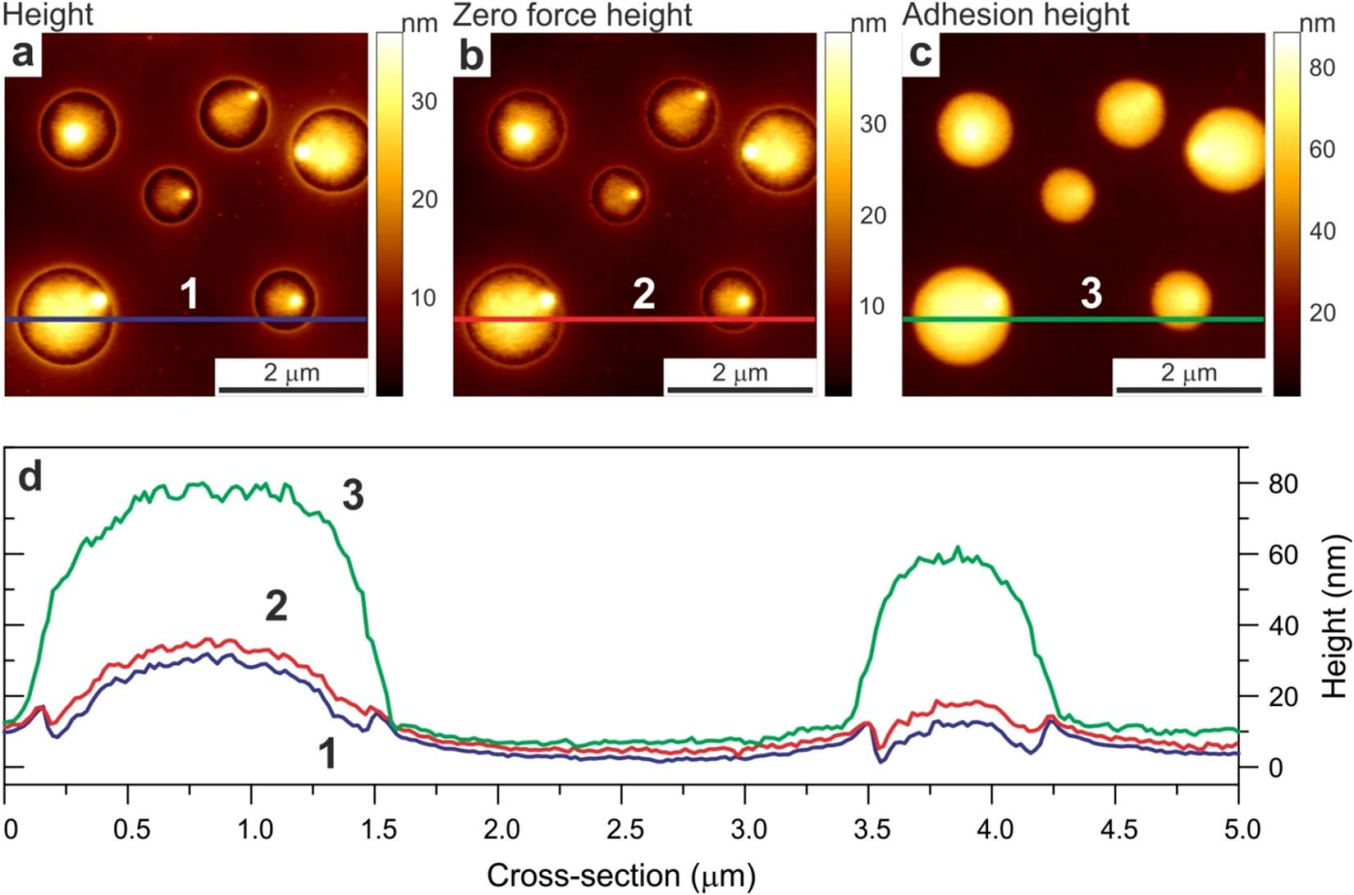
An example of simultaneous imaging of three different heights: (**a**) regular, (**b**) zero force, and (**c**) adhesion heights. The cross-sections lines shown in the images are presented in panel (**d**).

**Figure 7:**
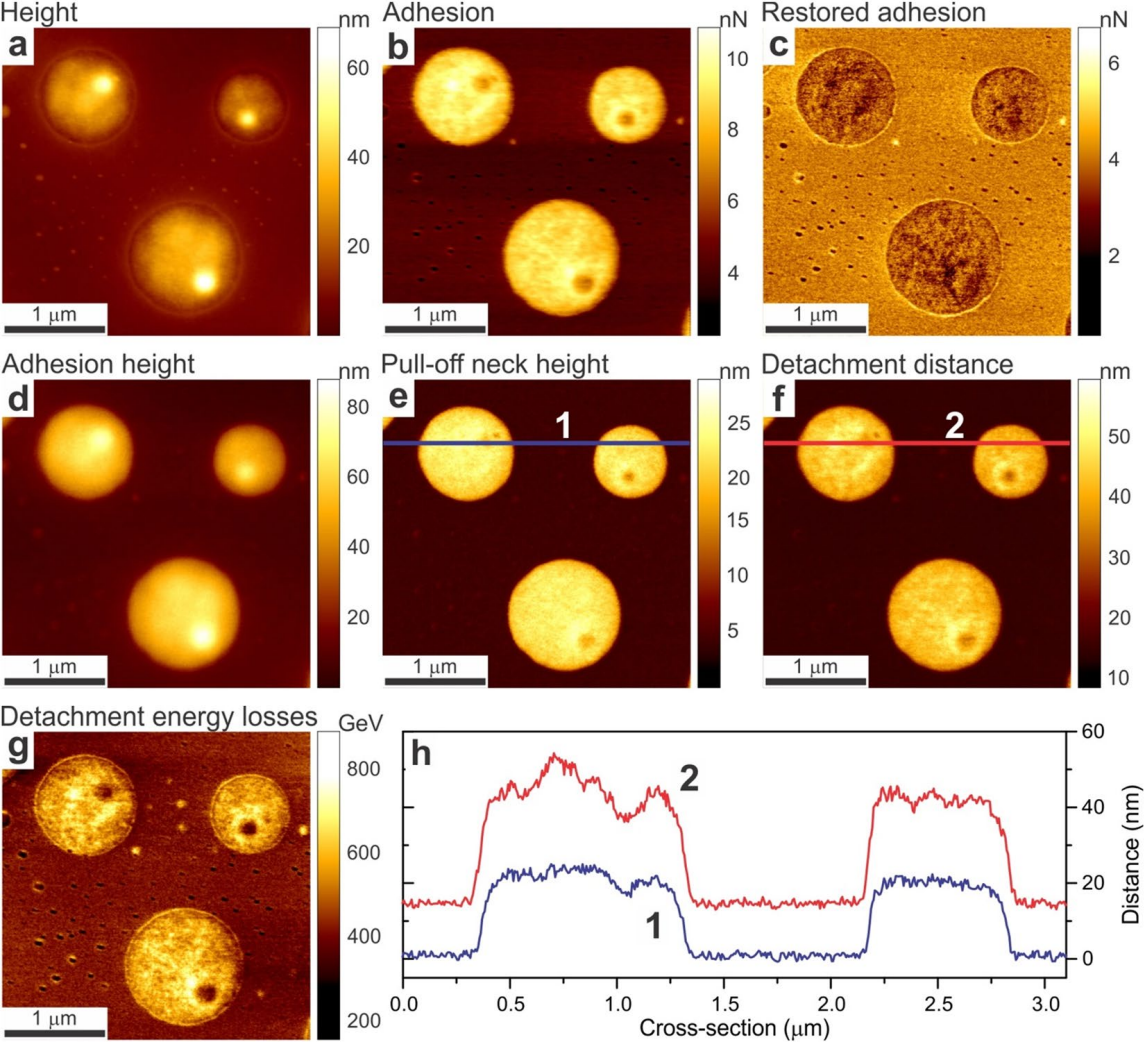
An example of all channels of ringing mode collected on a Bruker calibration sample, a blend of two polymers, low density polyethylene (DLPE) and polystyrene (PS). Regular height (**a**) and adhesion (**b**) images are shown for comparison. (**c**) The restored adhesion, (**d**) adhesion height, (**e**) pull-off neck height, (**f**) detachment distance, and (**g**) detachment energy losses are shown. (**h**) Comparison of cross-sections of the pull-off neck height and detachment distance showing a substantial difference between these two channels.

To demonstrate the difference between these two heights, we imaged a standard Bruker sample used for calibration of HarmoniX AFM mode, a blend of low density polyethylene (LDPE) and polystyrene. Figure 6 shows the images of such sample, simultaneously recorded regular, adhesion, and zero force heights. The latter is the height of the sample recorded in the moment when the deflection of the cantilever crosses zero. We do not claim here this channel as new because it has already been introduced in some commercial AFMs. If we assume zero force height as the profile of undeformed surface, one can clearly see a larger deformation on the load force (regular height) in the areas occupied by LDPE (round features seen in the images) compared to the deformations in between (the areas occupied by polystyrene). It is interesting to see a large relative pull-off deformation of LDPE imaged with the adhesion height. Comparing these results with the data shown in Fig. 7, one can see that the pull-off deformation of LDPE almost entirely consists of the pull-off height, the height of the neck pulled off the LDPE surface by the action of the AFM probe.

In addition, we can note that the adhesion height demonstrates much higher resolution compared to the regular height images. In the case of easily deformable surface features, this can be clearly seen by comparing Fig. 2e,f just visually. This is because the height at the moment of the adhesion is measured when the probe is pulling the surface rather than deforming it. A detailed analysis of the surface features measured in both adhesion and regular height modes is compared in detail in Supplementary note 4. In a particular example analyzed in that note, the adhesion height shows at least 3x better spatial resolution that the regular height image. The reason is due to different area of contact, which is much smaller for the adhesion height compared to the regular height. The latter is defined as the position of the sample at the moment of maximum load force, i.e., the maximum area of contact. The disconnection height is defined by the pull-off contact radius which is much smaller (and the lateral resolution is limited mostly by noise in this case).

#### Pull-off neck height and Disconnection distance

These two channels are novel, and do not have direct comparison with the regular sub- resonance tapping channels. The pull-off neck height allows seeing the distributions of heights of pull-off necks between the AFM probe and sample surface created by the adhesive action of the probe. The disconnection distance presents unique information about the size of the molecules weakly attached to the AFM probe during the final disconnection process. As an example, this data channel can demonstrate the size of glycocalyx layer molecules covering the surface of the cell (Fig. 3c).

#### Comparison of all channels together

To demonstrate we present all newly introduced channels together for the standard Bruker polymer blend of LDPE and polystyrene. Figure 7 shows these five channels together with the standard height and adhesion channels for the reference. One can see a substantial difference between adhesion and restored adhesion as was discussed above. The difference between heights where already discussed before. We would like to attract your attention to the difference between the pull-off neck height and detachment distance. The cross-sections of these values along the same line are shown in panel (h). One can see that these two physical channels are rather independent, having different values and spatially uncorrelated within the same polymer phase.

It is interesting to elaborate on the differences between regular and restored adhesions. For example, Fig. 2 shows rather similar qualitative behavior of both adhesions. At the same time, these two adhesion channels shown in Fig. 7 demonstrate rather dramatic difference. Although the difference is quite expected because Fig. 7 shows the image of two different materials, whereas Fig. 2 shows essentially the same (though multilayered) material, let us focus on the nature of the differences observed on PS and LDPE polymers in Fig. 7. To explain the difference between restored and regular adhesions observed on these polymers, let us recall that the difference between these two channels comes from residual stretching molecules which are still stuck to the AFM probe after the point of reaching adhesion (pull-off) force, point D of Fig. 1. This starting disconnection point is defined by the condition of mechanical balance between the force attracting the AFM probe to the sample surface and the force of the AFM cantilever trying to disconnect the probe from the surface. Mechanical disconnection starts when the gradients of these forces become equal. Rupturing of residual (physical) bonds between sample molecules and the AFM probe is happening between points D and E of Fig. 1. It happens when the gradient of the attraction force, which is produced by these molecules, is less than the spring constant of the AFM cantilever. Because both polymers in Fig. 7 were imaged with the same AFM probe, the big difference between adhesion and restored adhesion observed between PS and LDPE polymers is explained by a relatively large difference of molecular interaction of these polymers with the AFM probe. This difference can also be seen in rather different detachment distances (the size of the stretched molecules) shown for PS and LDPE polymers in Fig. 7e and f, respectively. Specifically, the AFM probe pulls LDPE molecules up to ~40–50 nm, whereas only ~15 nm on PS polymer.

## Discussion

Here we showed a powerful extension of sub-resonance tapping modes of atomic force microscopy, ringing mode, which allows obtaining more robust and novel information about the surface of biologically relevant materials, cells and polymers. Ringing mode is based on the use of ringing signal of the AFM cantilever, which occurs right after disconnection of the AFM probe from the sample surface. This signal has been either filtered or ignored in regular sub-resonance tapping modes. We demonstrated that one can obtain new information about the sample surfaces by processing this signal. Five new channels of information can be recorded simultaneously in addition to the ones existing in sub-resonant imaging: restored adhesion, disconnection energy loss, adhesion height, pull-off neck height, and disconnection distance. When applied to soft materials, in particular cells, imaging in these channels can be up 20x faster, with less artifacts, and with higher spatial resolution.

Based on these advantages, we envision multiple application of this ringing mode in soft matter research, in particular, in biomedical applications. For example, as was demonstrated^21,22^, the adhesion maps can be used to detect cancer cells with high accuracy. However, it takes about 90 min to get one adhesion map when using regular PeakForce Tapping mode. Because a reliable diagnosis cannot be done based on a single cell, the imaging of multiple cells is expected. This would require excessively long time, which preclude considering this AFM imaging as a diagnostic modality. 20x acceleration in ringing mode may make this method clinically promising. The images of the adhesion heights can provide details of the cell surface which cannot be seen in other height modes due to deformation of the sample by the probe during the imaging.

Another interesting application may be related to force spectroscopy^27–29^, a popular AFM application that allows measuring the strength of molecular bonds. The described ringing mode allows recoding directly the energy of rupture of molecular bonds occurred due to the contact between the AFM probe and sample surface, for example, if the molecules are attached to a relatively rigid surface (the disconnection energy loss channel). The disconnection distance can show the size of the molecules grafted on the sample surface. These highly promising applications will be studied in future works.

## Methods

### AFM setup

Measurements were performed using an Icon and Bioscope Catalyst AFMs (both by Bruker-Nano Inc) equipped with NanoScope V controller. Bioscope catalyst was mounted on the top of Nikon TE2000U microscope. Standard Bruker ScanAsyst-Air AFM cantilevers were used to obtain all images shown in this work. The images shown in this work were acquired while scanning in air using standard air probe holders. Exactly the same procedure is applicable for scanning in water. The only difference will by using of Bruker ScanAsyst-Fluid AFM probes. To implement ringing mode, one needs to collect unfiltered (raw) signals from AFM. Details of such implementation are described in Supplementary note 1.

### Preparation of cells

A375 human melanoma skin epithelial cells (ATCC CRL-1619) were fixed with Karnovsky fixative as described, in ref.^22^. Specifically, the cells were washed twice with PBS, drained, and left in Karnovsky fixative solution for 12 hours at 4 °C. Cells were then washed with PBS, kept two hours at room temperature, and washed again. PBS was changed for deionized (DI) water and kept in water for 12 hours at 4 °C. Cells were then washed with DI water twice and prepared for freeze-drying. After fast freezing in a freezer with temperature −40 °C–80 °C, the frozen cells in water were transferred to the freeze dryer and kept until complete drying (4–5 hours). The freeze-dried cells were imaged right after extraction from the freeze dryer. Alternatively, the dried cells can be stored in a desiccator (up to 2–3 weeks) and imaged later. The dried cells were imaged with AFM with no further preparation.

### Preparation of skin flakes

Cuderm Dark (carbon filled) adhesive tape strips (D-squame skin indicator D200, CuDerm, Corp.) were used for skin corneocyte collection^30^. This tape was advantageous for both force measurements (allows easy to dissipation of electrical charges which may create additional long-range forces, suppressing the AFM signal) and for optical prescreening, see the Imaging Protocol section. The collected flakes were first pre-screened with the help of an optical microscope. This allowed avoiding imaging regions with a high aspect ratio. The flakes were then imaged with AFM with no further preparation. This study of human skin flakes did not constitute human subject research as defined by the IRB office of Tufts Medical Center and Tufts University Health Sciences.

### Imaging Protocol

Measurements presented in this work were performed in air (though the same can be repeated in water provided enough adhesion between the AFM probe and surface). All AFM probes were calibrated before imaging to determine the deflection sensitivity, spring constant, resonance frequency, and quality factor (Q) of the AFM cantilever in air. The standard calibration of the deflection sensitivity was performed against clean Si wafer. The spring constant of the cantilevers (~0.35–0.5 N/m) was measured by using the thermal fluctuation method^31^. The resonance frequency and quality factor of the cantilever were estimated by performing a power spectral density analysis of the cantilever’s thermal oscillations using build-in function in the Bruker Nanoscope AFM software. The resonance peak was fitted with the simple harmonic oscillator model: $A(\omega )={A}_{0}+{A}_{\omega 0}{\omega }_{0}^{2}/\sqrt{{({\omega }_{0}^{2}-{\omega }^{2})}^{2}+{\omega }_{0}^{2}{\omega }^{2}/{Q}^{2}}$, where *A*_0_ is the baseline amplitude (white noise baseline), *A*_ω0_ is the amplitude at zero frequency, ω_0_ is the center frequency of the resonant peak and *Q* is the resonant peak quality factor. Obtained parameters were used to calculate the restored adhesion, eq. 1. It should be noted that the Q factor should be measured within the vicinity of the sample because its value substantially influenced by the proximity of the sample surface. Figure S3 demonstrates such a dependence, showing a constancy of the Q factor when the tip – surface distance is smaller than approximately 600 nm (note that this is the maximum amplitude of tapping that is used in this work). Such behavior is virtually independent on the particular sample material.

Prior to the imaging, all samples were mounted on standard 25 × 75 mm glass slides (Fisherbrand Superfrost Plus Microscope Slides, Fisher Scientific). 15 mm steel coins (AFM Specimen Discs, Ted Pella, Inc.) were pre-glued to the bottom of the glass slides with epoxy resin (Araldite, Bostik Ltd, Staffordshire, UK). The steel coins allow using magnetic holders to fix the slides on the Bruker Icon AFM stage. Humidity dependence experiments were performed inside the Bruker Icon AFM enclosure chamber. Humidity was controlled by balancing moisture created by an ultrasonic evaporator (TT-AH002, TaoTronics,) and dry nitrogen gas (NI UHP300, Airgas) inside the chamber. This allows controlling relative humidity in a range of 20–80%.

The imaging procedure was optimized by following the standard protocol for PeakForce Tapping Bruker imaging mode. The load force during PeakForce Tapping imaging was varied from 250 pN up to 10 nN. In the majority of experiments, the load force of 1 nN was used in order to have a robust contact between the AFM probe and the sample. The imaging can be done at the load force below 250 pN as soon as the contact between the sample and the AFM probe can be achieved at each point of the image.

For each sample, up to five standard AFM and three new ringing mode data channels (chosen out of five available) were collected. The scan size was varying from 100 × 100 nm up to 25 × 25 μm, although the minimal and maximal scan size can be limited only by the parameters of the AFM scanner. The digital resolution in this work was chosen between 256 × 256 and 1024 × 1024 pixels. 0.5 Hz scan rate was used for most of the images collected in this work. This was done to preserve information collected with standard AFM channels, which demonstrate visible deterioration at higher speeds (see for example, Supplementary Figure S1). Only the new ringing mode data channels can be imaged with the speed up to 5 Hz. The vertical ramping frequency was set to 1 kHz. The vertical peak-peak amplitude was varying between 50 to 600 nm. It should be noted that the cantilever amplitude must be big enough to allow complete disconnection between the AFM probe and the sample for each pixel of the map. This is particular important for soft and sticky samples or if long molecules are presented on the surface.

### Data collection and processing

All computations were performed using LabVIEW programming language (National instruments). Offline processing was partially done with the help of the SPIP software package (Image Metrology, Inc.). The unfiltered AFM data signals can be collected by using various methods described in Supplementary note 1. The data can further be processed with a variety of external data processing software, for example Matlab, Mathcad or LabVIEW. It is possible to process the recorded signal in real time by using real-time data processing hardware. Some basic manipulations (adding and subtraction of images, multiplication by a constant) can be done off-line within the regular Bruker NanoScope software, or more advanced software processing (for example SPIP). It should be noted that the statement about faster work of ringing mode is applicable no matter if the collected data were processed off-line or online. The processing algorithms are described in the main text.

### Data availability

The datasets, AFM images scanned and analysed during the current study are available from the corresponding author on reasonable request.

## Electronic supplementary material

Supplementary info
