# Supplementary material for: Nanoscale compositional mapping of cells, tissues, and polymers with ringing mode of atomic force microscopy
Source: Sci Rep. 2017 Sep 19;7:11828. doi: 10.1038/s41598-017-12032-z (PMC5605548; doi:10.1038/s41598-017-12032-z)
Supplement: Supplementary file 1 — Supplementary info [file 41598_2017_12032_MOESM1_ESM.pdf]

Supplementary materials

for

Nanoscale compositional mapping of cells, tissues, and polymers with ringing mode of atomic force microscopy

M. E. Dokukin[1], I. Sokolov, [1,2,3,]*

[1]Department of Mechanical Engineering, [2]Department of Biomedical Engineering, [3] Department of Physics, Tufts University, Medford, MA, USA

Contents

Supplementary figure S1 2
Supplementary figure S2 3
Supplementary figure S3 4
Supplementary Note 1: Notes on implementation ringing mode 5
Supplementary note 2: Contribution of water layer (capillary interaction) to the energy disconnection loss when imaging in air 6
Supplementary note 3: Relation between the Disconnection energy loss and Dissipation energy parameter 10
Supplementary note 4: Demonstration of higher resolution of the adhesion height compared to the regular height 11

Supplementary figure S1

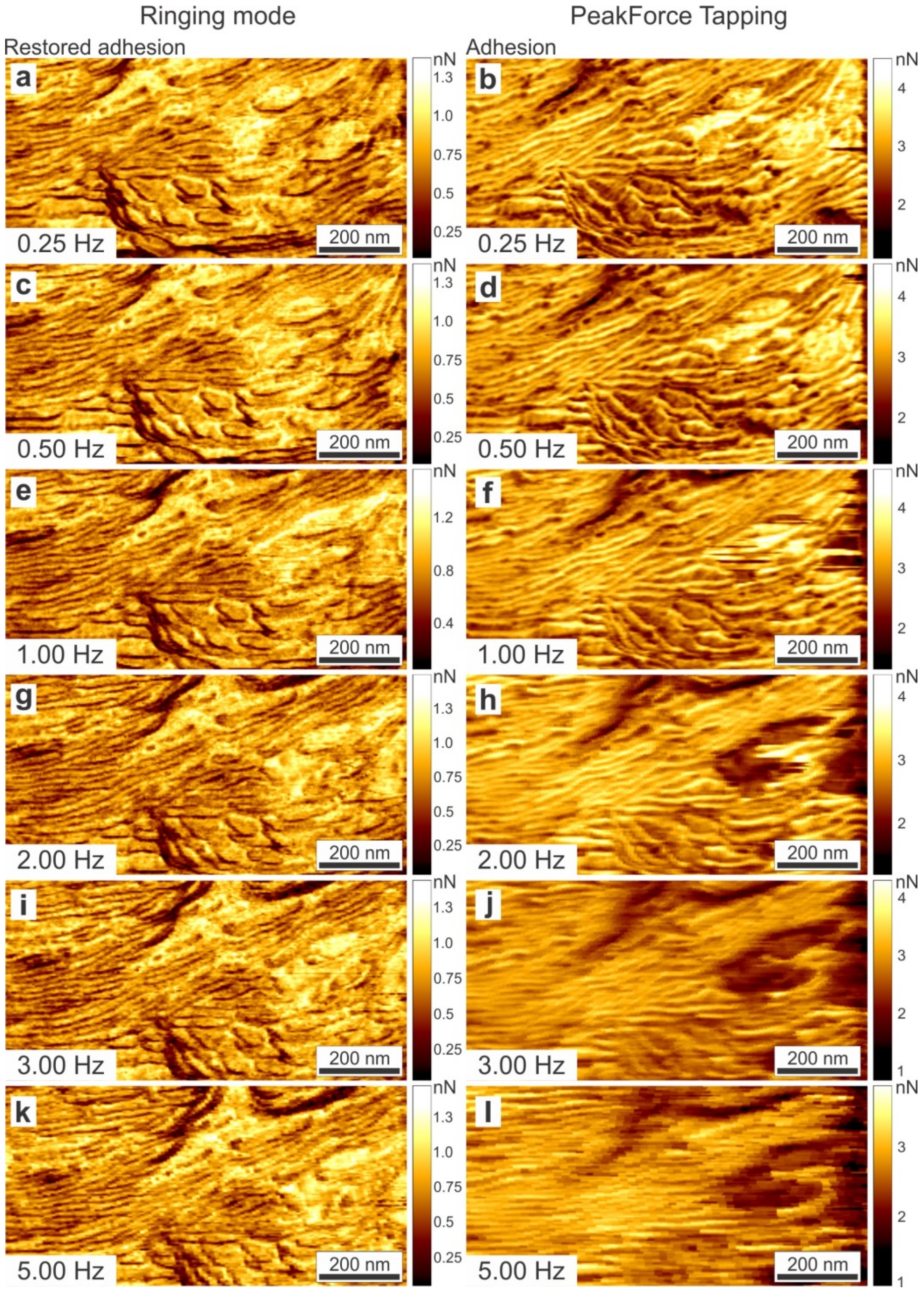

Supplementary figure S1 | Comparison of the restored adhesion maps recorded in the new ringing mode (the left panels, a,c,e,g,i,k) with the regular adhesion maps (the right panels, b,d,f,h,j,l) recorded with PeakForce QNM mode. Maps of polystyrene-polycaprolactone composite polymeric material are shown. Lamellar structures of the polycaprolactone polymerare clearly seen in ringing mode even at the 5Hz scan speed.

Supplementary figure S2

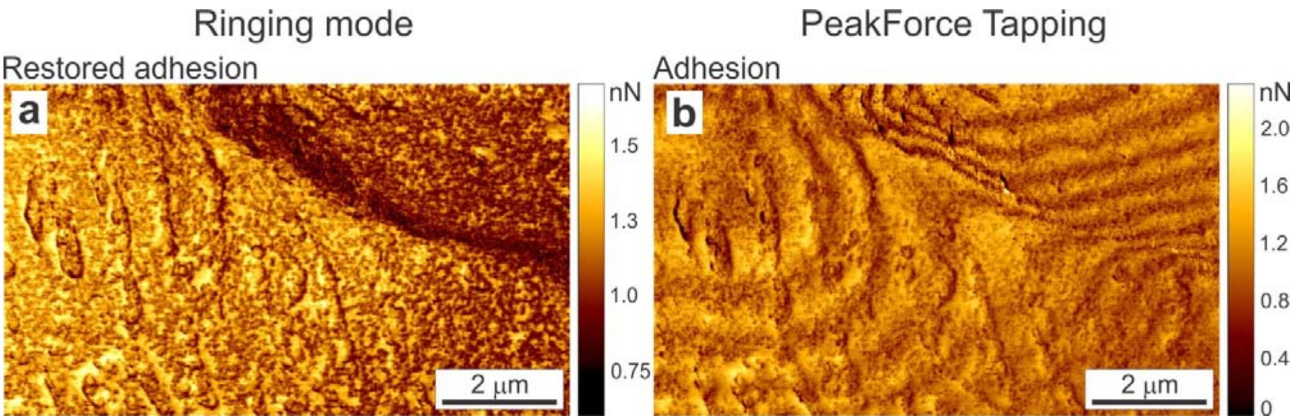

Figure S2 | An example of 6x10 um^2 cell surface simultaneously imaged with (a) the ringing mode (the averaged restored adhesion data channel), and (b) PeakForce QNM mode (the adhesion data channel). The wavy artifacts (due to laser interference) are clearly seen in the adhesion channel, but not seen in the new restored adhesion channel. The scan rate is 0.1 Hz.

Supplementary figure S3

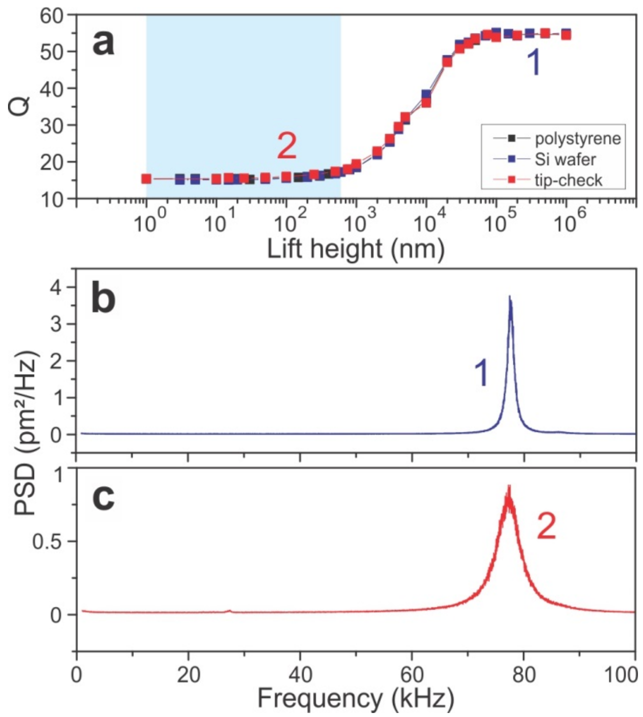

Figure S3 | (a) Dependence of the quality factor on the distance between the AFM probe and sample surface (lift height). Examples of the recorded power spectral density (b) far away from the surface, region 1, and (c) close to the surface, region 2. Ringing mode works within the distances of region 2.

Supplementary Note 1: Notes on implementation ringing mode

Two atomic force microscopes were used to implement ringing mode described in this work, Bioscope Catalyst and Icon (both by Bruker-Nano, former Veeco, Inc.). Both microscopes were controlled by NanoScope V controller, and were capable of running the sub-resonant tapping mode, PeakForce QNM.

It should be noted that the latest versions of NanoScope V controller (64-bit controller) are capable of recording force curves at each pixel of the entire image. The older version (32-bit controller) allow researchers to record only the force curves for one line of pixels in the image.

The filtering of the deflection signal coming from the AFM cantilever is controlled through the software. Because of multiple versions of the NanoScope software, there are different options available to the user. If the software allows to switch off the low pass filter (or at least, to move the threshold frequency well above the resonance frequency of the cantilever), the unfiltered signal can be recorded by just choosing this option.

The unfiltered signal can also be recorded directly using "high-speed data acquisition", another option within the NanoScope software. Finally, the NanoScope hardware allows recording the raw unfiltered signal by means of any suitable external data acquisition card. It is recommended to check with the manufacturer if the user's software is capable or can be upgraded for the direct unfiltered signal recording.

The recorded force curves can be processed with any external data processing software, such as Matlab, Mathcad, LabVIEW. It is possible to process the recorded signal in real time by using real-time data processing hardware, for example, National Instruments real-time and FPGA solutions. Some basic manipulations (adding and subtraction of images, multiplication by a constant) can be done off-line within the regular NanoScope software, or more advanced software processing (for example, SPIP, ImageMetrology).

Supplementary note 2: Contribution of water layer (capillary interaction) to the energy disconnection loss when imaging in air

Capillary force between two spherical contacts can be estimated as follows [1-4]:

$$F_c = 2\pi\gamma_L R^*\left(2c - \frac{h}{r}\right) \approx 2\pi\gamma_L R^*\left(\cos\Theta_1 + \cos\Theta_2 - \frac{h}{r}\right), \quad \text{(S1)}$$

where γ_L – interfacial tensions between liquid and vapor (for water γ_L=0.072 N/m); $R^* = R_{probe} \cdot R_{surface} / R_{probe} + R_{surface}$ is the effective probe-surface contact radius; *c* is the mean cosine of the contact angles: $c = 1/2\left[\cos\left(\Theta_1 + arcsin\left(l/R_{probe}\right)\right) + \cos\Theta_2\right] \approx \left(\cos\Theta_1 + \cos\Theta_2\right)/2$ (here we assumed that $l \sim R_{probe}$); *l* is the azimuthal radius of the liquid meniscus; *h* – is the distance between two spheres and r is the meridional meniscus radius: $r = -\lambda_K / \ln\left(P/P_0\right)$, where λ_K – so called "Kelvin length" $\lambda_K = \gamma_L V_m / RT$ and V_m is the molar volume of the liquid medium, (0.52 nm for water)[5,6], P/P_0 is a relative humidity (P_0 – is a dew point). Θ_1 and Θ_2 are the contact angles between the liquid surface and the AFM probe and the liquid surface and the sample respectively.

The energy required to break the capillary bridge, $W_{capillary}$, can be estimated by integrating the capillary force (eq. S1) over the distance between the height of the pull-off neck (h_{neck}) and the maximum possible height of the capillary bridge (h_{disc}):

$$W_{capillary} = \int_{h_{neck}}^{h_{disc}} 2\pi\gamma_L R^*\left(\cos\Theta_1 + \cos\Theta_2 - \frac{h}{r}\right)dh = \frac{\pi\gamma_L R^*}{r}\left(h_{neck} - r\left(\cos\Theta_1 + \cos\Theta_2\right)\right)^2, \quad \text{(S2)}$$

where $h_{neck} = \left(\pi^2 w_{adh}^2 R^* \Big/ \frac{64}{3} E^{*2}\right)^{1/3}$, here w_{adh} is the adhesion energy per unit area between the AFM probe and the sample surface and E^* is the reduced Young's modulus of the sample material: $E^* = E/\left(1 - v^2\right)$, where E is the sample Young's modulus and *v* the sample Poisson

ratio. Disconnection height h_{disc} (relative to non-deformed surface) can be estimated as

$$h_{disk} \sim -\lambda_K / \ln(P/P_0)(\cos\Theta_1 + \cos\Theta_2) = r(\cos\Theta_1 + \cos\Theta_2).$$

We will now demonstrate that the contribution of capillary interaction can be neglected in the case of the samples considered in our work (and further, for virtually any biological sample with reasonable surface properties, see the below). To estimate the contribution of the energy losses due to the breakage of capillary bridge, the probe and surface curvature radii as well as humidity and material stiffness are required. The radius of surface curvature (asperities) can be estimated from the AFM height images (the *adhesion* height was used because the disconnection energy losses are happening during disconnection from the *adhesion* point) of the sample surface. An example of such surface is shown for a specific sample of human skin flakes, Supplementary Fig.S4a. Doing the analysis of the sample cross-sections and feeding the radius of curvature (done with the help of SPIP software, see, [7] for detail), we can find that a typical radius of the asperity ranges in the limits of 20-200 nm, Supplementary Fig.S4b.

Using these data, we can plot the values of the energy losses for the found ranges of the radii of the surface curvature (asperities). Taking various probe radii, relative humidity, hydrophobicity (contact angle of air-material-water contact), and material elastic modulus, which are typical for biological materials, we can obtain the energy losses, Supplementary Fig.S5. These results can be compared with the measured disconnection energy losses (shown in figure 2c). One can see that the presented values are negligible in comparison to the values of the disconnection energy loss obtained for the skin flakes. (Here the experimental parameters used during scanning are the follow: probe radius ~8 nm, relative humidity – 45%, sample Young's modulus ~ 10 MPa and the contact angles are 110 and 22 for the skin flake and SiO_2 probe respectively.)

Furthermore, one can see that the energy losses due to the capillary interaction are quite small (compared to the observed values) for a broad range of parameters, and getting to be noticeable only for rather rigid materials and high humidity (Fig.S5d, c). Because typical biological materials are rather soft, the capillary bridge contribution is presumably negligible for the majority of bio materials. If not, it can be made negligible by decreasing humidity to the required level. In general, the graphs shown in Fig. S5 can be used to estimate the energy losses due to the breakage of the capillary bridge between the AFM probe and sample surface.

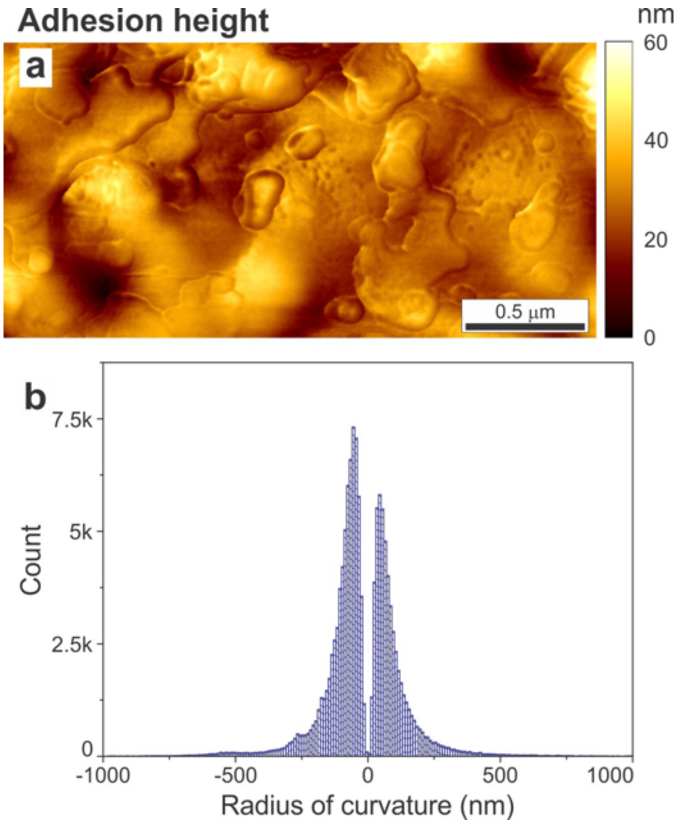

Supplementary Figure S4 | Examples of skin flake surfaces used to estimate the radii of surface curvature (asperities). The adhesion height images are used. a) An AFM image of the adhesion heights. b) A statistical distribution of the radii of curvature of the surface shown in panel a).

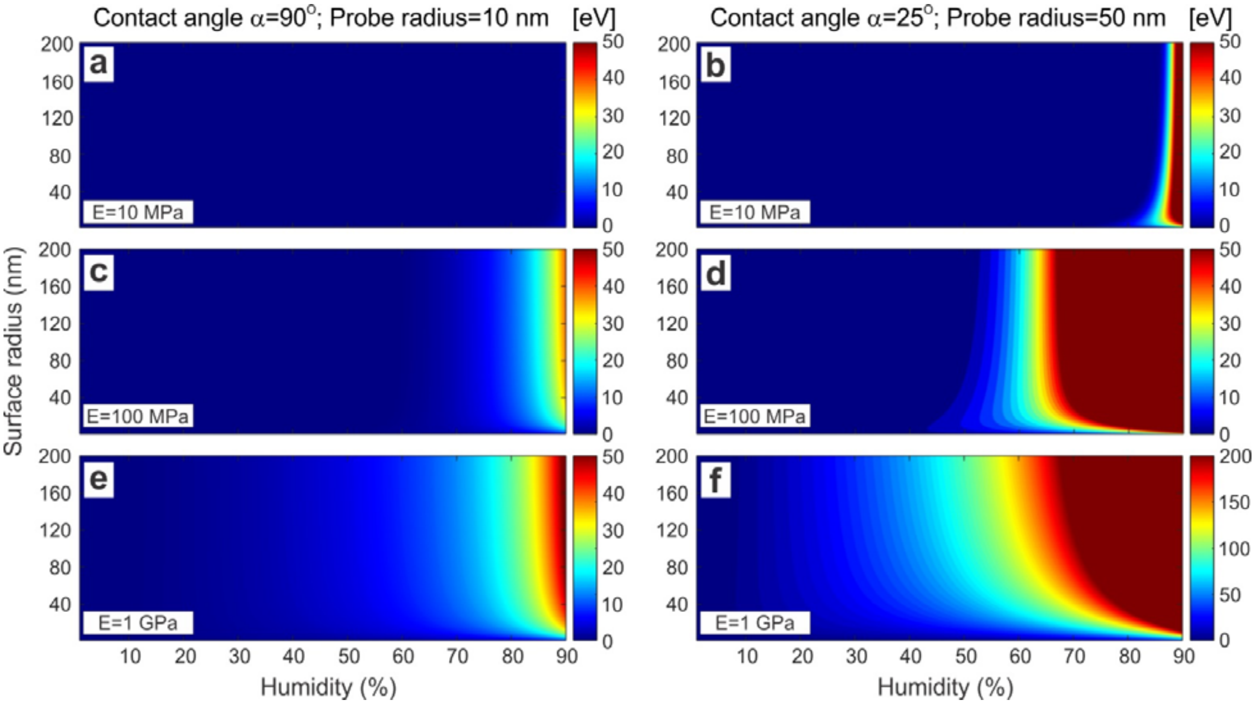

Supplementary Figure S5 | Theoretical estimations of the energy losses due to the presence of capillary bridge between the AFM probe and sample surface.

Supplementary note 3: Relation between the Disconnection energy loss and Dissipation energy parameter

The easiest way to show connection between the Disconnection energy loss and Dissipation energy parameter is to present them graphically, Fig.5 of the main text. By its definition, dissipation energy is mainly defined by the viscoelastic response of the sample during the probe indentation. In contrast, the disconnection energy loss parameter is defined only by the energy lost by the cantilever starting from the moment of pool-off until complete disconnection of the probe from the sample surface. For example, this can be due to long molecules weakly adhering to the AFM probe, which are still being stretched by the AFM probe after pulling off.

It is interesting to note that the value of disconnection energy loss is not correlated with adhesion. Fig.S6 demonstrates such a case when the force curves show the same adhesion but substantially different disconnection heights (and consequently, the different disconnection energy losses because the larger the disconnection height, the more energy is lost during the disconnection, see also Figure 1b of the main text for graphical explanation). These particular force curves are recorded on a polystyrene sample, which surface was processed with plasma, which presumably creates a number of dangling carbon bonds. Thus, Fig.S6 most likely shows the differences in the presence of such carbon bonds. This will be studied in future works.

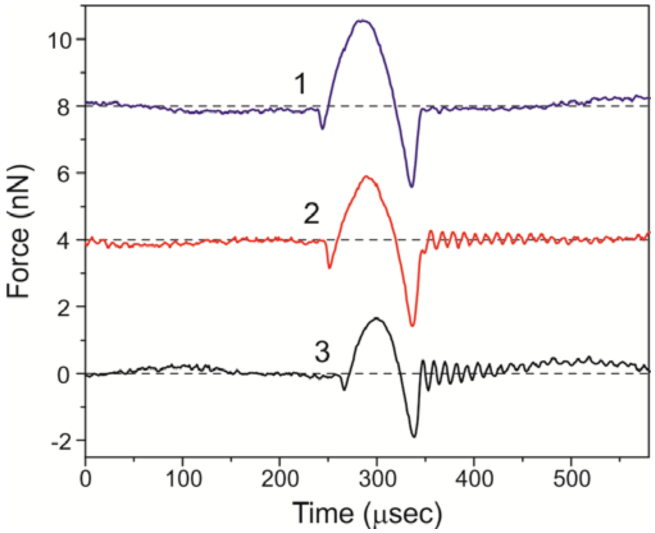

Supplementary Figure S6 | Example of the same adhesion but different disconnection heights (disconnection energy losses).

Supplementary note 4: Demonstration of higher resolution of the adhesion height compared to the regular height

The (spatial) resolution of newly introduced adhesion height channel is expected to be higher compared to the regular height mapping. This is because the resolution is defined by the area of contact between the AFM probe and sample. The regular height is defined at the moment of maximum indentation. The contact at the moment of measuring the adhesion (pull-off) height is defined by the diameter of the neck connecting the AFM probe and sample at the moment of pull off contact. Obviously, the area of such neck is always smaller than the contact area at the moment of maximum indentation.

Supplementary Figure S7 shows the comparison between the adhesion height (introduced in our ringing mode) and regular height (recorded with the commercial PeakForce QNM mode). One can clearly see more details in the adhesion height image (Fig.S7a) compared to the regular height (Fig.S7b). Cross-sections of the same region taken for the adhesion and regular heights, Fig.S7c allow seeing much higher level of details in the adhesion height cross-section (line 1). A more statistical confirmation of higher resolution of surface features seen in the adhesion height is presented in Figs. S7d-g. The radii of curvature of surface features presented in the height images (Fig.S7a,b) are plotted. One can see that the most frequent values are the radii observed in the adhesion height are about three times smaller than the ones in the regular height (~100nm vs 300nm, respectively). One finds virtually the same proportion by comparing the radii of the smallest features seen in the adhesion heights (Fig.S7f, radii of the smallest features are ~20nm) and the regular heights (Fig.S7g, the smallest features have radii ~50-70nm). Thus, one can conclude that the adhesion height shows ~3x better spatial resolution that the regular height image in this particular example. This difference can be even more dramatical depending on the probe radius and the maximum load force, which mostly define the resolution of a conventional height image.

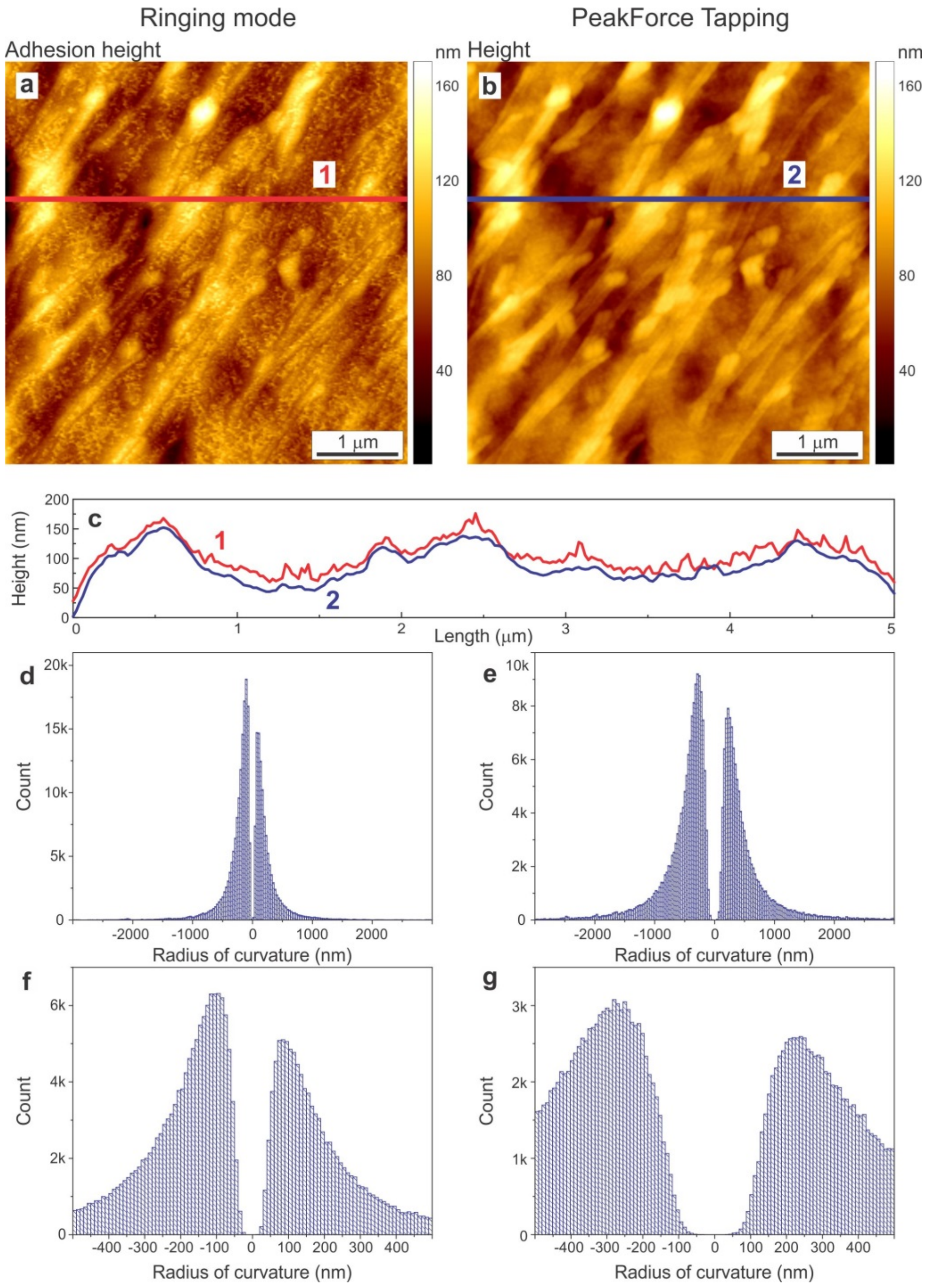

Supplementary Figure S7 | Height images of A375 cell surface. (a) the adhesion height recorded in ringing mode, (b) the regular height recorded with PeakForce QNM. (c) a typical cross-section of the surface obtained from the adhesion height image (line 1) and the regular height (line 2). (d, e) the radii of the curvature of the adhesion and regular heights, correspondingly. (f,g) the zoomed versions of panels (d,e), respectively, to demonstrate most frequent values of the curvature radii observed in the height images.

Supplementary References

1. Butt, H.-J. & Kappl, M. *Surface and Interfacial Forces*. (Wiley-VCH, 2010).
2. Pitois, O., Moucheront, P. & Chateau, X. Liquid bridge between two moving spheres: An experimental study of viscosity effects. *Journal of Colloid and Interface Science* **231**, 26-31, doi:DOI 10.1006/jcis.2000.7096 (2000).
3. Pitois, O., Moucheront, P. & Chateau, X. Rupture energy of a pendular liquid bridge. *Eur Phys J B* **23**, 79-86, doi:DOI 10.1007/s100510170084 (2001).
4. Rabinovich, Y. I., Esayanur, M. S. & Moudgil, B. M. Capillary forces between two spheres with a fixed volume liquid bridge: Theory and experiment. *Langmuir* **21**, 10992-10997, doi:10.1021/la0517639 (2005).
5. Israelachvili, J. N. *Intermolecular and surface forces*. 3rd edn, (Academic Press, 2011).
6. Kohonen, M. M. & Christenson, H. K. Capillary condensation of water between rinsed mica surfaces. *Langmuir* **16**, 7285-7288 (2000).
7. Dokukin, M. E., Guz, N. V., Gaikwad, R. M., Woodworth, C. D. & Sokolov, I. Cell surface as a fractal: normal and cancerous cervical cells demonstrate different fractal behavior of surface adhesion maps at the nanoscale. *Physical Review Letters* **107**, 028101 (2011).
